# Advances in Epicardial Access for Ventricular Tachycardia Ablation: From Historical Techniques to Carbon Dioxide Insufflation

**DOI:** 10.31083/RCM47009

**Published:** 2026-04-22

**Authors:** Koji Higuchi, Jakub Sroubek, Justin Lee, Pasquale Santangeli

**Affiliations:** ^1^Cardiac Electrophysiology and Pacing Section, Department of Cardiovascular Medicine, Cleveland Clinic, Cleveland, OH 44195, USA

**Keywords:** ventricular tachycardia, epicardial access, CO_2_ insufflation, right atrial appendage

## Abstract

Epicardial access is often required for effective catheter ablation of ventricular tachycardia (VT) originating from the epicardium, especially in patients with non-ischemic cardiomyopathy, arrhythmogenic right ventricular cardiomyopathy (ARVC), and cardiac sarcoidosis. Traditional subxiphoid access using large-bore needles remains effective but carries substantial procedural risks, including right ventricular (RV) puncture, coronary artery injury, and injury to intra-abdominal organs. These risks are amplified in patients with prior cardiac surgery, obesity, and distorted anatomy. To mitigate these challenges, several technical advancements have been introduced, including the needle-in-needle (micropuncture) technique and the SAFER approach using RV angiography during apnea. However, these methods do not fully overcome the inherent limitation of minimal separation between the pericardial layers at the time of pericardial puncture. Carbon dioxide (CO_2_) insufflation into the pericardial space is a recently developed technique that can temporarily separate the parietal and visceral pericardium for safer epicardial access. Coronary venous exit for CO_2_ has demonstrated safety and efficacy, as confirmed in the multicenter Epi-CO_2_ Registry. Further advances include the use of radiofrequency (RF)-assisted trans-right atrial appendage (RAA) perforation for CO_2_ insufflation. In this comprehensive review, the advancement of epicardial access is discussed from the early era to contemporary techniques, especially regarding CO_2_ insufflation, including its pitfalls and the future direction of this technique.

## 1. Introduction 

Catheter ablation has become a cornerstone in the management of ventricular tachycardia (VT), especially in structural heart disease, where antiarrhythmic drugs often fail. In patients with ischemic cardiomyopathy (ICM), most VTs originate from endocardial scars. However, epicardial substrates are very common in non-ischemic cardiomyopathy (NICM), arrhythmogenic cardiomyopathy, such as arrhythmogenic right ventricular cardiomyopathy (ARVC), and cardiac sarcoidosis. Therefore, the epicardial substrates have influenced epicardial mapping strategies [1, 2]. Delineating substrate location using late gadolinium-enhanced cardiac magnetic resonance imaging (MRI) [3], unipolar voltage mapping [4], and ECG criteria [5] have been utilized more frequently in understanding epicardial substrates. The combination of precise imaging and safe access techniques has significantly expanded the therapy options for effective VT ablation.

## 2. Traditional Subxiphoid Epicardial Access and Risks

**Fig. 1.**
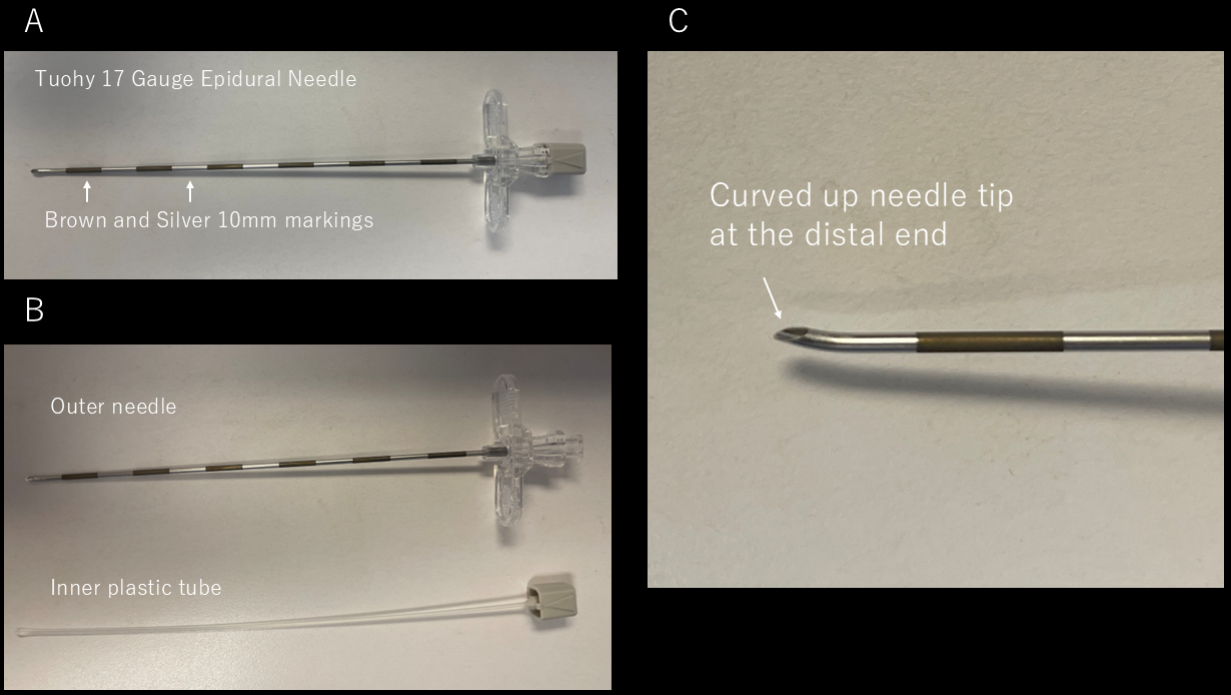
**Tuohy needle**. (A) Picture of Tuohy 17 Gauge Epicardial needle. The needle has brown and silver markers at a distance of 10 mm. (B) Picture of the outer needle and the inner plastic tube. (C) Magnification of the needle tip, showing the curved-up shape at the distal end.

The technique for percutaneous “dry” pericardial access was first systematically described by Sosa *et al*. in 1996 [6]. Their technique laid the foundation for modern epicardial VT ablation and remains the standard approach for epicardial access. Standard epicardial access involves direct subxiphoid puncture with a large-bore needle with a curved end (also known as Tuohy or Pajunk needle, originally developed for epidural anesthesia and later adapted for epicardial access) under fluoroscopy (Fig. 1). Due to the high level of patient discomfort associated with a puncture, general anesthesia is recommended to ensure the patient’s immobility which reduces diaphragmatic movement, and allows for optimal breath-hold during the critical moment of pericardial puncture. Furthermore, preparation for emergency blood transfusion and cardiothoracic surgery backup is strongly recommended.

After induction of general anesthesia, the abdomen is prepped and draped in a sterile fashion, and fluoroscopic guidance is initiated, usually in the anteroposterior (AP) projection. A combination of the AP and left anterior oblique (LAO) 90 ° projections is often used to navigate the needle trajectory and avoid the posterior abdominal structures. If the view of the LAO 90 ° is not visible due to the patient’s body habitus (this is often encountered in patients with obesity and/or elevated diaphragm), the LAO and right anterior oblique (RAO) projections are used.

**Fig. 2.**
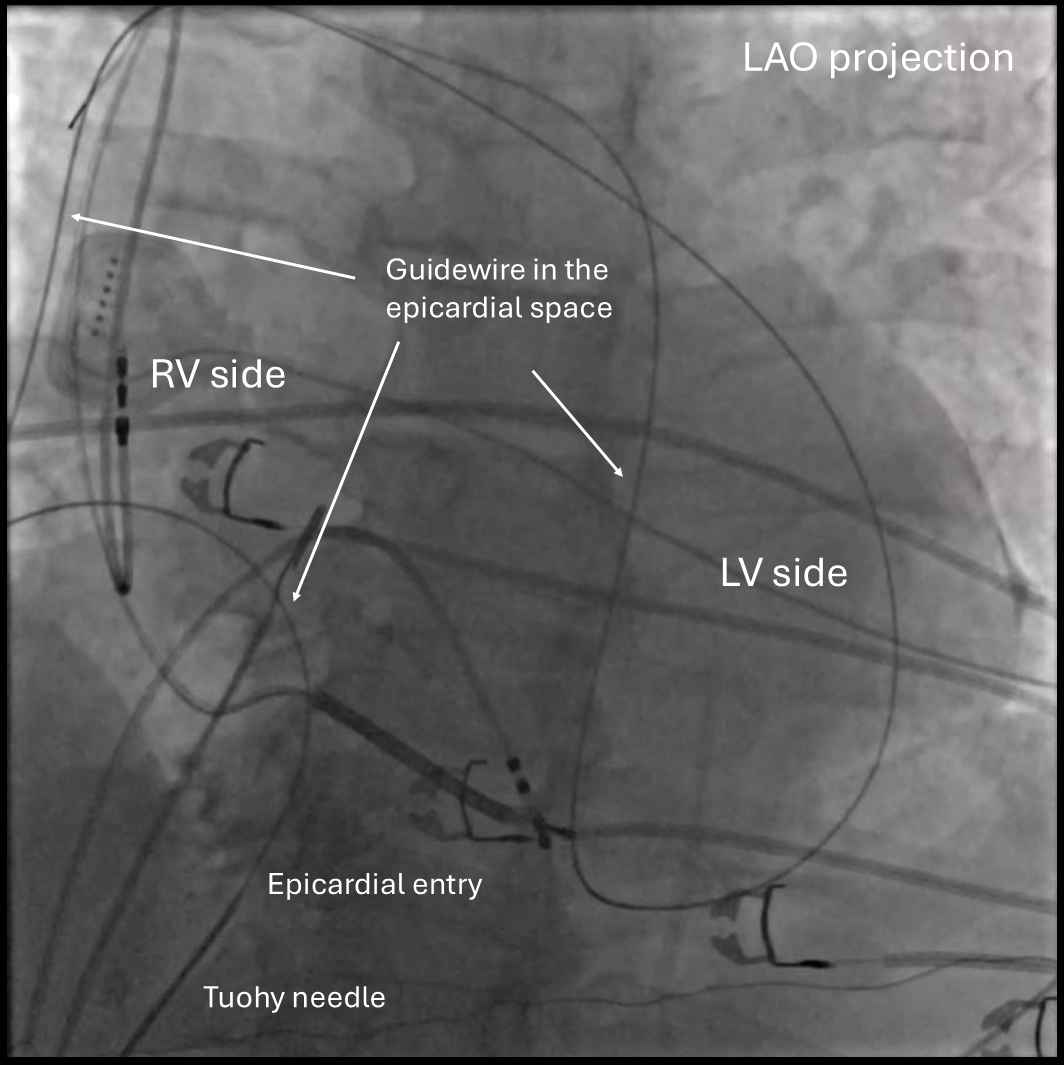
**Confirmation of wire placement in the epicardial space**. Confirming the guidewire crossing from the right side to the left side of the heart is generally recommended by fluoroscopy with an LAO projection. Note that the wire goes only within the cardiac silhouette. LAO, left anterior oblique; RV, right ventricle; LV, left ventricle.

The puncture site is typically 1–2 cm inferior to the xiphoid process and slightly leftward. A skin incision is made to facilitate the passage of the large needle. While manually depressing the abdomen, the large-bore needle is advanced through the subcutaneous tissues and abdominal wall muscles toward the left shoulder, maintaining a shallow angle relative to the skin. Contrast was originally used only to confirm the needle placement once the needle is in the epicardial space. However, this was later modified to the use of contrast intermittently with fluoroscopic monitoring to observe a “tenting” of the parietal pericardium just before the puncture as well. Just before perforating the parietal pericardium, resistance may be felt as a “pulsatile feeling” from the needle. Once a “popping feeling” is felt, which indicates a puncture of the pericardium, contrast is injected to confirm the needle is in the intrapericardial space. A 0.035-inch guidewire is advanced under fluoroscopic visualization. It is important to confirm that the guidewire is not in the endocardium by fluoroscopy before sheath placement. Confirming the guidewire crossing from the right to the left side of the heart is strongly recommended using fluoroscopy with LAO projection (Fig. 2). One should also confirm that the wire is not outside the cardiac silhouette to make sure the wire is not in the pleural space. An intracardiac echocardiogram is also helpful to confirm that the guidewire is not in the RV. Following the wire placement, a long steerable or fixed sheath is inserted into the pericardial space. Anticoagulation is typically withheld until successful sheath placement to reduce the risk of hemorrhagic complications.

Despite its utility, this procedure involves inherent risks, including RV puncture, hemopericardium, coronary artery injury, phrenic nerve injury, and damage to abdominal organs, which can be up to 7.5% even in high-volume centers [7, 8, 9]. These risks are further elevated in patients with prior pericarditis or cardiac surgery due to adhesions in the pericardial space. The traditional approach relies heavily on anatomical intuition and two-dimensional fluoroscopic projections. This often limits spatial accuracy and increases procedural difficulty in patients with distorted thoracic anatomy or obesity.

## 3. Anterior vs. Posterior Approach 

The anterior approach is the most commonly used technique in contemporary practice. In this method, the needle is advanced in a relatively anterior direction, typically aimed toward the left shoulder. Under fluoroscopic guidance, the needle is introduced at a shallow angle relative to the skin. This trajectory generally allows entry into the pericardial space just anterior to the RV. The anterior approach is often preferred in patients with normal or mildly rotated cardiac anatomy, as it facilitates more direct access to the anterior, lateral, and apical surfaces of the left ventricle.

In contrast, the posterior approach employs a steeper needle trajectory directed inferiorly and posteriorly toward the diaphragmatic surface of the heart. This path typically advances the needle deeper into the abdomen, passing posterior to the liver and occasionally in proximity to bowel loops, before reaching the pericardial space.

The most important difference between these two approaches lies in their complication profiles. With the anterior approach, the primary risks include inadvertent RV puncture, given the proximity of the entry site to the RV surface, as well as injury to the left internal mammary artery (LIMA) if the needle trajectory passes too close to the sternum.

**Fig. 3.**
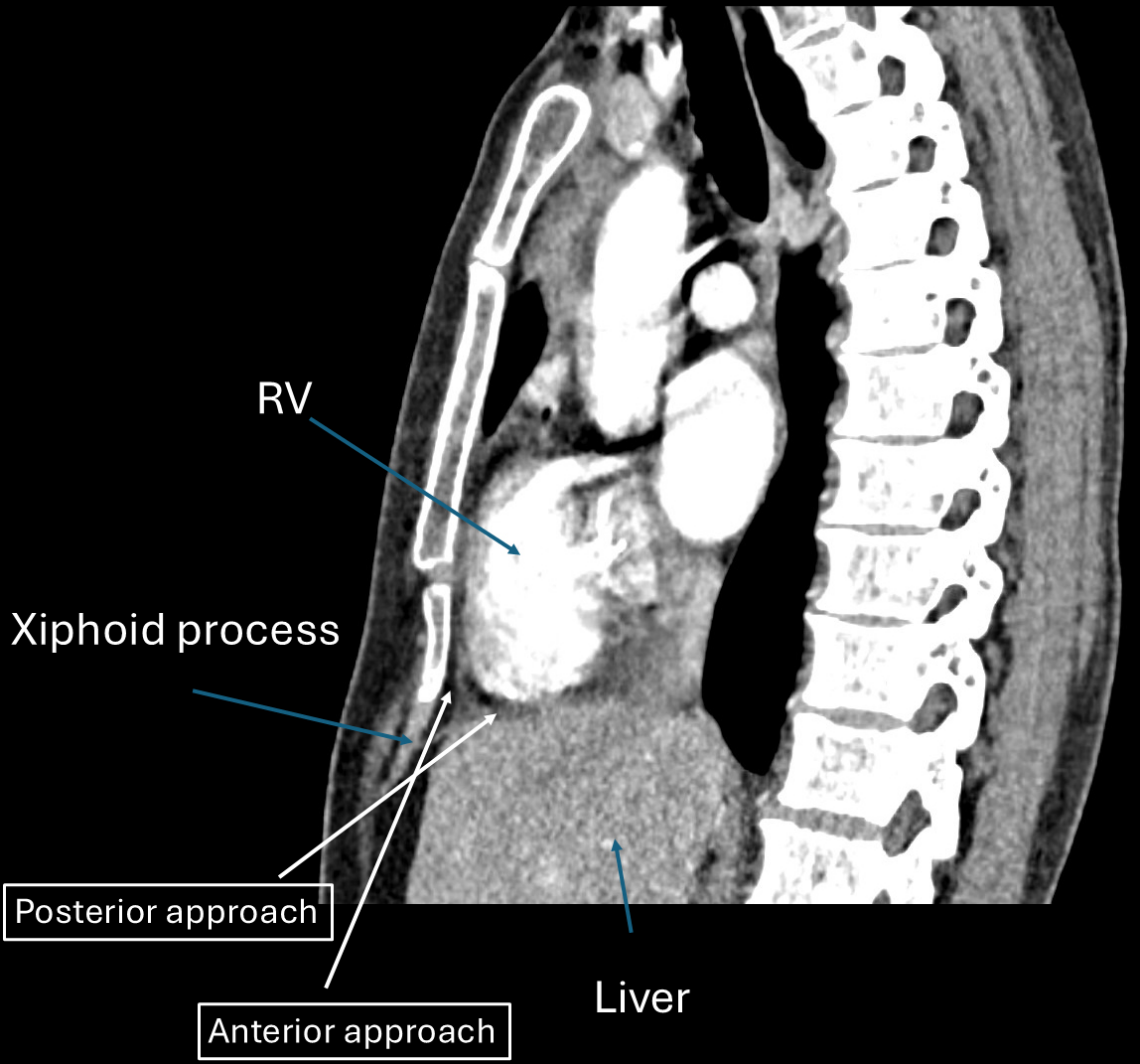
**Anterior and posterior approach**. This figure demonstrates the difference in angle and entry for both anterior and posterior approaches. Typically, skin entry is lower in the anterior approach as opposed to the posterior approach. RV, right ventricle.

On the other hand, the posterior approach carries a higher risk of serious non-cardiac complications. As the needle passes through the diaphragm and into the abdominal cavity, it is in close proximity to vital organs, including the liver, bowel, colon, and inferior epigastric arteries. Case series and retrospective analyses have identified hepatic laceration, intra-abdominal hemorrhage, and bowel perforation. These might be potentially life-threatening complications, especially when hypotension occurs during the procedure without evidence of cardiac tamponade. While the posterior approach can be effective in specific scenarios, such as when the anterior space is obliterated due to adhesions from prior open-heart surgery [10], it is generally considered less favorable in routine practice due to its higher risk profile [11]. Although rare, when epicardial ablation needs to be considered as an approach to access the epicardial substrate for atrial fibrillation, a posterior approach is required to reach the posterior left atrium and roof, where additional ablation is typically needed [12]. Fig. 3 demonstrates the difference in angle and entry for both anterior and posterior approaches. Typically, skin entry is lower in the anterior approach as opposed to the posterior approach, which requires the needle angle to be adjusted to be shallower.

## 4. Alternative Approach to Mitigate the Risk of Complications

### 4.1 Needle-in-Needle Technique

**Fig. 4.**
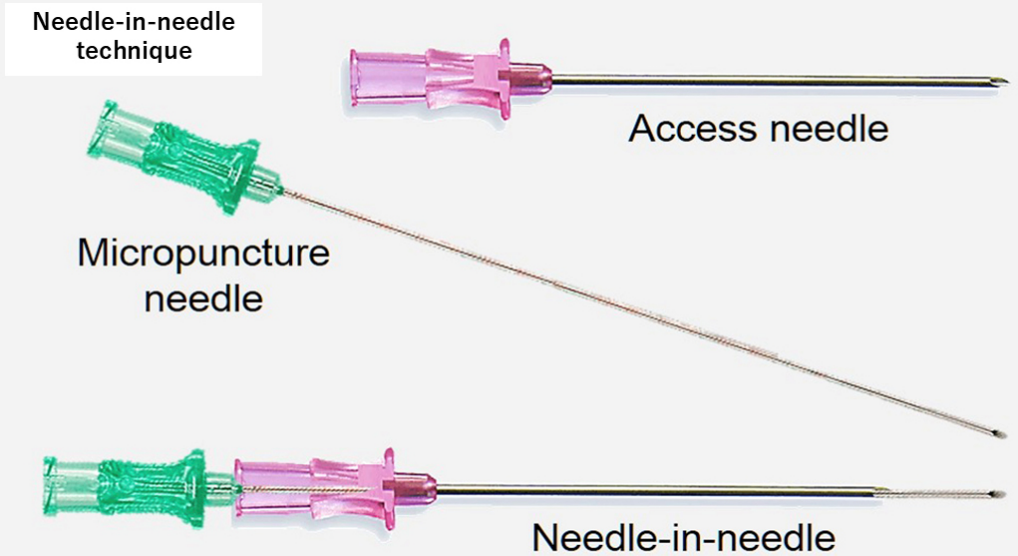
**Needle-in-needle technique**. This figure shows how to assemble two needles for the needle-in-needle technique. This figure was modified from the original figure of Aryana *et al*. [15].

The use of micropuncture needles (typically 21G) together with a large-bore needle was first described by Kumar *et al*. [13]. Their technique involved direct percutaneous subxiphoid access using a long 21G needle supported by a short 18G needle that serves as an introducer. This combination, known as the “telescoping technique”, allows the outer 18-gauge needle to guide the micropuncture needle through the subcutaneous and muscular layers, which is particularly useful in patients with obesity. Once the 18G needle is advanced close to the pericardium, the 21G needle is used to puncture the pericardium. After successful entry into the pericardial space, confirmed by contrast injection, a 0.018-inch guidewire is advanced and subsequently exchanged for a standard sheath using a transitional dilator. The safety of this approach was confirmed by a large multicenter observational study [14]. However, the sensation of “popping” into the epicardial space, which is often the sign of pericardial space entry, might be less due to the small needle size. Thus, the risk of RV puncture is still high, although it is still deemed less risky than large-bore needles. The needle assembly for this technique is shown in Fig. 4 (Ref. [15]).

### 4.2 Sustained Apnea for Epicardial Access With Right Ventriculography (SAFER Epicardial Approach)

**Fig. 5.**
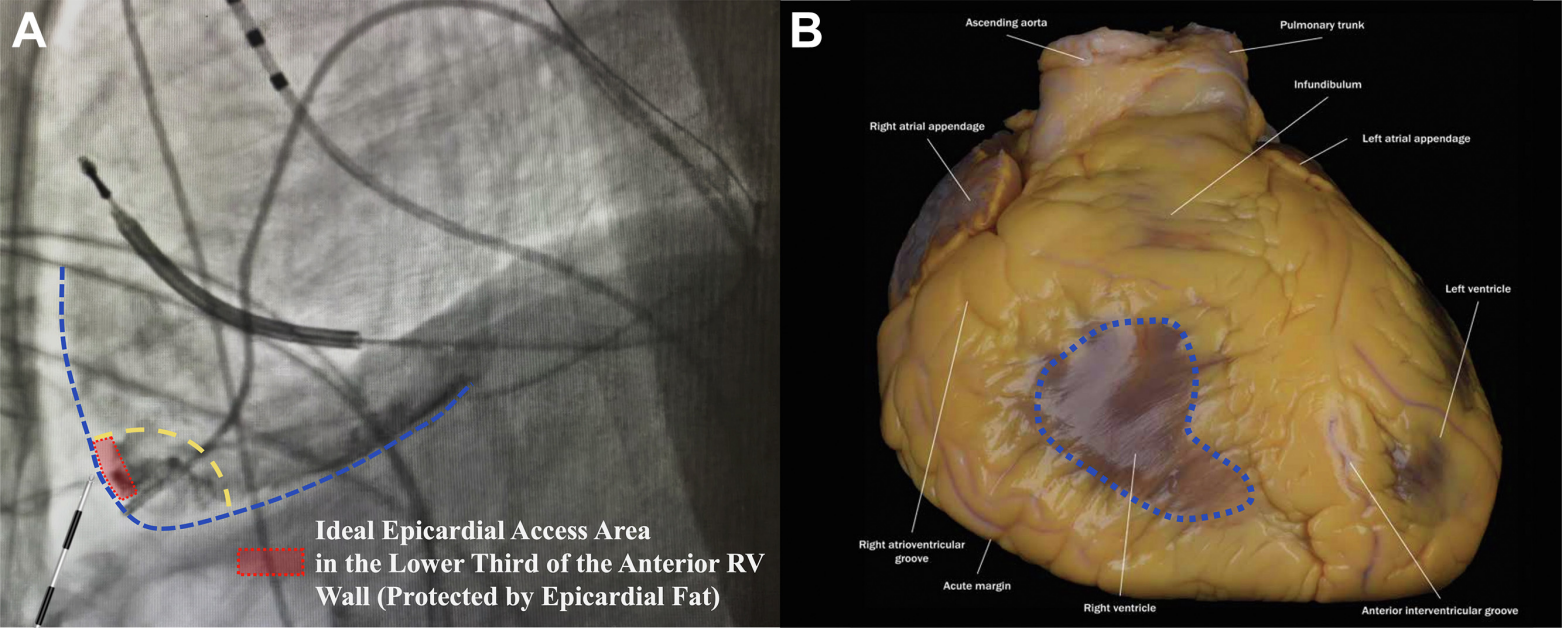
**Sustained apnea for epicardial access with right ventriculography (SAFER epicardial approach)**. (A) Delineation of the ideal pericardial puncture site in LL fluoroscopic projection at the lower third of the right ventricular (RV) wall (dotted red rectangle), leftward to the interventricular septum. This area is usually protected by epicardial fat in most patients. (B) Illustration of the area of the pericardium that should be avoided as it is not protected by epicardial fat (dotted blue area). This figure was modified from the original figure of Romero *et al*. [16].

This technique was introduced by Romero *et al*. [16], who provided additional refinements to enhance the safety and reproducibility of the standard method. An important aspect of this approach is respiratory control with sustained end-expiratory apnea and RV angiography. RV angiography with a 5 Fr pigtail catheter was performed in both anteroposterior (AP) and left lateral (LL) fluoroscopic projections during apnea to minimize cardiac and diaphragmatic motion, which further delineated the RV border and guided the needle entry. Once the ideal needle trajectory was evaluated, a 17G Tuohy large-bore needle was advanced under LL projection while manually compressing the abdomen to flatten the needle trajectory and displace the intra-abdominal organs. The lower third of the RV anterior wall was targeted for the entry point with the anterior approach, where it is typically insulated by epicardial fat and is free from major coronary vessels. The other aspects of this technique are the same as the conventional approach. The SAFER technique emphasizes its wide availability by utilizing commonly available tools, although it remains unclear to what extent each step of the procedure, and in particular, RV angiography, adds value and safety to the access procedure compared to standard “dry” puncture while maintaining the patient in apnea. Fig. 5 (Ref. [16]) depicts the ideal epicardial puncture location (lower third of the anterior RV wall) with RV angiography.

## 5. Carbon dioxide (CO_2_) Insufflation via Coronary Sinus for Safer Epicardial Access

**Fig. 6.**
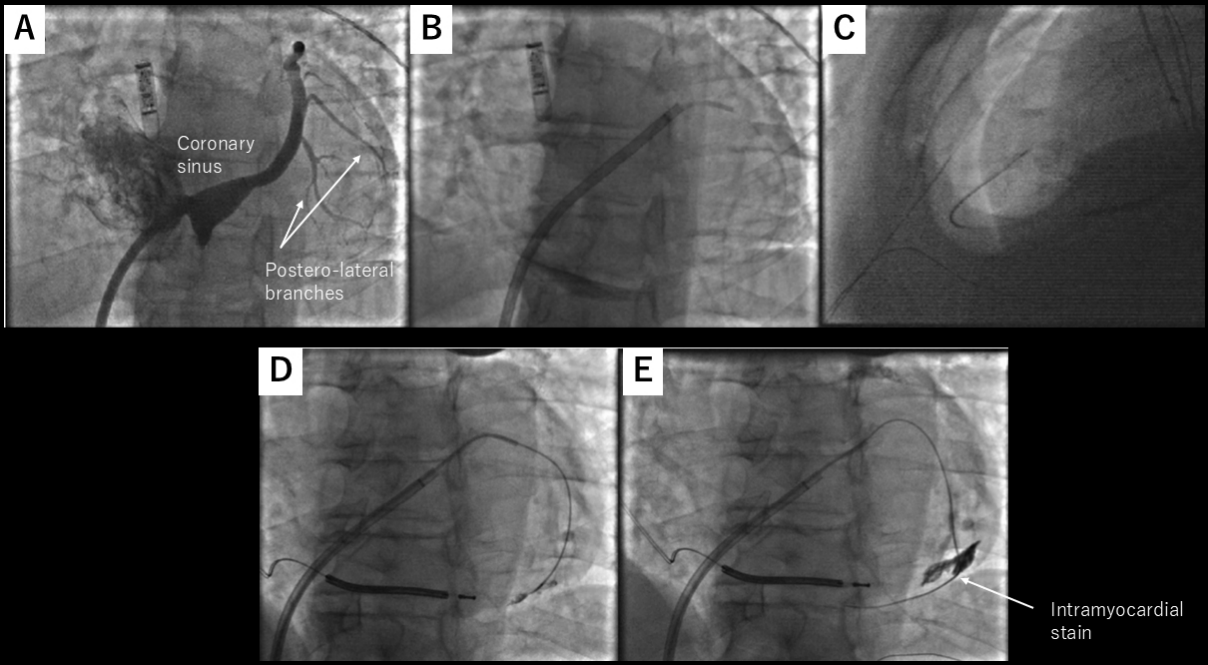
**CO_2_ insufflation from the coronary sinus exit**. (A) Left anterior oblique view of the coronary sinus. The target vein is one of the lateral veins, as highlighted by the arrows. (B) One of the lateral veins was selected by diagnostic JR4 catheter, and microcatheter was advanced over a coronary guidewire. (C) Microneedle entered the pericardial space, which has been insufflated with CO_2_. (D,E) Coronary wire may stray into an intracardiac vein. These figures were modified from the original figure of Silberbauer *et al*. [18].

Although several techniques have been implemented to minimize the risk of inadvertent injury to the heart or adjacent structures, such as the needle-in-needle approach [13, 14] or the use of sustained apnea with RV angiography, [16] we still encounter a fundamental challenge: typically, there is minimal or no anatomical separation between the parietal and visceral pericardium at the time of needle entry. To overcome this issue, the use of CO_2_ insufflation into the pericardial space was first introduced by Greenbaum *et al*. [17]. The rationale behind the use of CO_2_ is that it is non-flammable, colorless, highly soluble in blood, and rapidly absorbed by tissues, making it an ideal gas for transient anatomical separation. CO_2_ rises anteriorly in the pericardial space when a patient is in the supine position, providing a temporary air gap between the anterior RV and the pericardium, aiding the operator in confirming adequate separation of the visceral and parietal pericardium by fluoroscopy. Greenbaum *et al*. [17] used the right atrial appendage (RAA) as an exit to the epicardial space with the back end of a stiff coronary guidewire to perforate the RAA, which was limited by a high failure rate due to the inability to effectively perforate the thick RAA myocardium. Therefore, as first described by Silberbauer *et al*. [18], this was later modified to perforate the distal end of the coronary venous branch as an exit to the pericardial space. A high-tip-load coronary guidewire (0.014-inch) is used to perforate a distal lateral or anterolateral coronary vein branch supported by a deflectable sheath inserted in the coronary sinus. A microcatheter is advanced into the pericardial space over this wire once a coronary guidewire is in the epicardial space. Then, after a small contrast injection into the pericardial space, CO_2_ is manually insufflated into the pericardial space. This allows for precise subxiphoid access with a 22G microneedle (which later replaced the initially used 18G Tuohy needle to avoid rapid gas loss and pericardial collapse) (Fig. 6A–C, Ref. [18]). Severe hemopericardium is usually rare, as verified by the Epi-CO_2_ registry [19], as opposed to the conventional “dry” puncture approach, which reported RV puncture in 3–17% and significant pericardial bleeding in up to 9% of cases. The safety is further confirmed in a single high-volume referral center [8] as well as a mid-volume referral center [20].

This technique is also useful for detecting pre-existing pericardial adhesion by injecting contrast once pericardial access is obtained by a microcatheter, as described in the Epi-CO_2_ registry [19]. Also, on a case report basis, this technique was reported to be effective in patients with epicardial adhesion [10]. However, there is still insufficient evidence for the use of this technique for severe epicardial adhesion.

One important thing to be aware of when using a coronary sinus as an exit is that a coronary wire may stray into an intracardiac vein, as described in the initial report (Fig. 6D,E) [18]. Also, the risk of dislodging the coronary sinus lead for cardiac resynchronization therapy with a defibrillator (CRT-D) needs to be taken into account due to the complexity of the maneuver in the coronary sinus.

## 6. Radiofrequency Assisted Intentional Right Atrial Appendage Puncture for CO_2_ Insufflation

Although CO_2_ insufflation via the coronary veins has been shown to be safe and effective, its relatively lengthy and multistep nature is still a limitation. In addition, patients who require epicardial VT ablation often have CRT-D in place, which makes it more difficult to safely cannulate a suitable coronary venous branch without compromising the existing coronary sinus pacemaker lead.

**Fig. 7.**
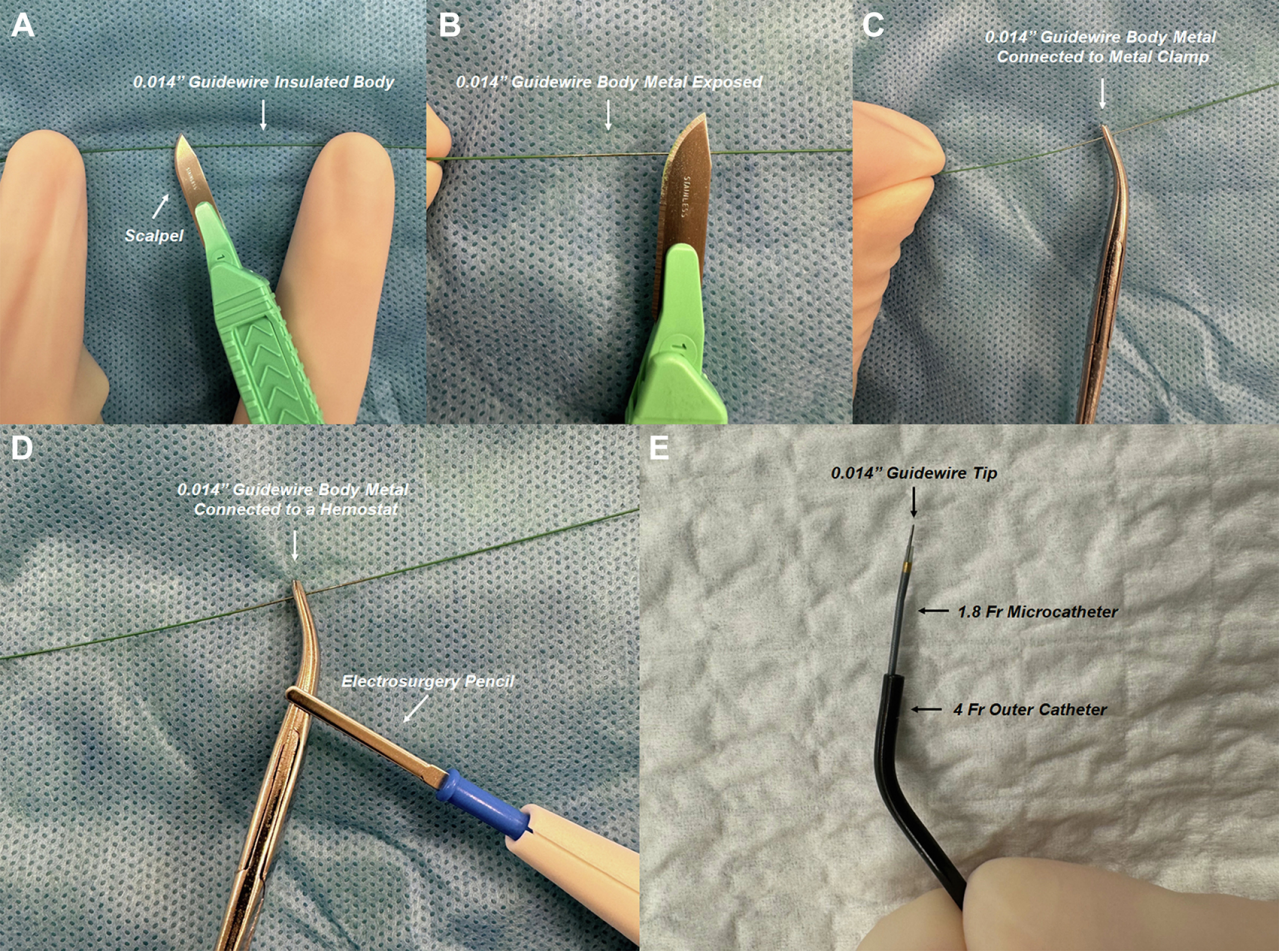
**RF guidewire-microcatheter assembly**. (A,B) A surgical blade is used to shave off the insulation from the proximal end of a stiff 0.014-inch guidewire (exposed metal tip and insulated body). (C,D) The proximal exposed metal end of the guidewire is connected to an electrosurgery pencil with a hemostat clamped to the guidewire to ensure constant contact during RF application. (E) The custom RF guidewire-microcatheter assembly is shown. These figures were from the original figure of Santangeli *et al*. [21].

**Fig. 8.**
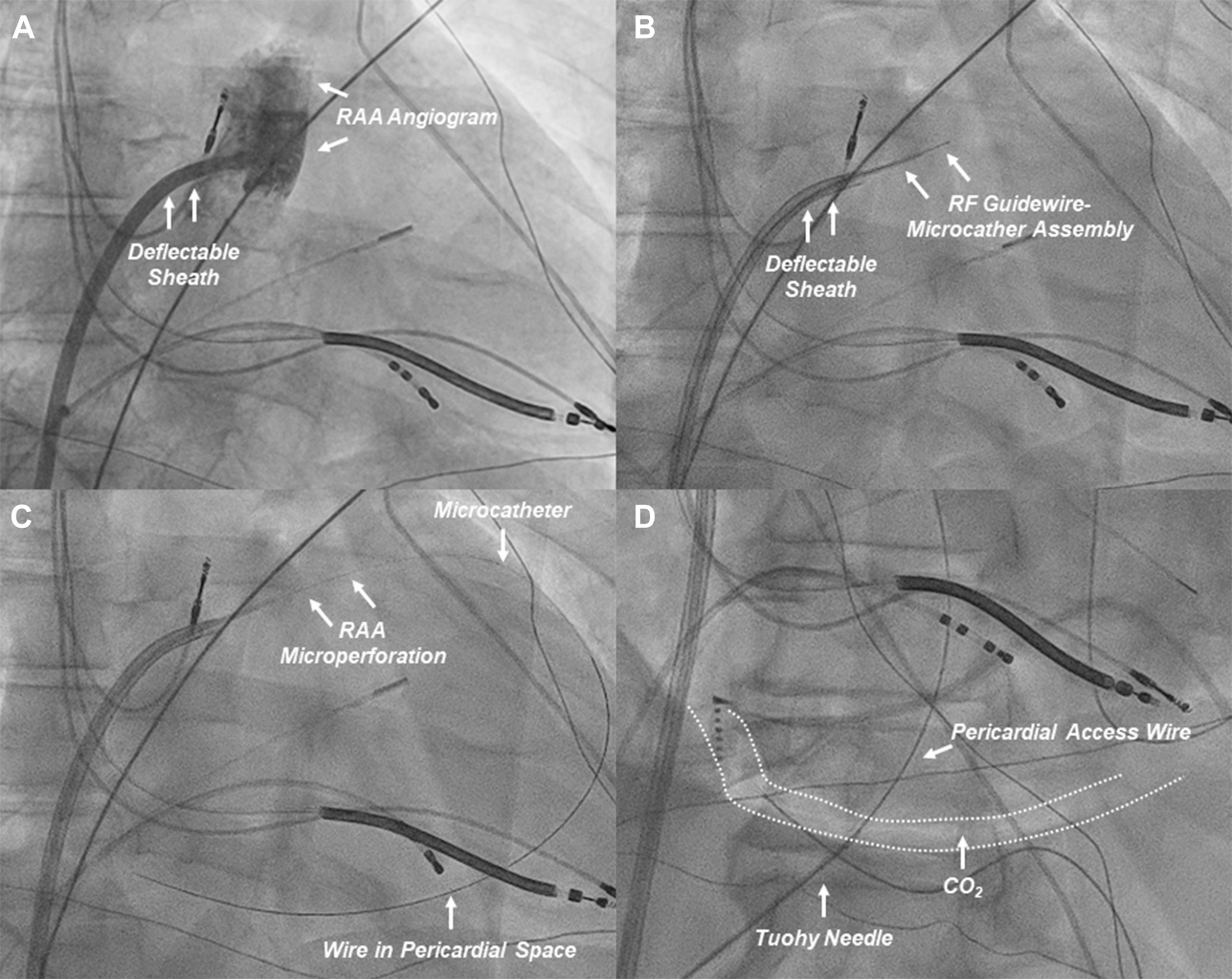
**Procedural workflow of RAA perforation for CO_2_ insufflation**. (A) An RAA angiogram (right anterior oblique projection) is obtained from a deflectable sheath positioned at the base of the RAA. (B) The custom RF guidewire-microcatheter assembly is advanced to obtain contact with the target RAA wall (anterior aspect of the distal third of the RAA). (C) The RAA wall is perforated to advance the wire and microcatheter to the pericardial space for CO_2_ insufflation. (D) After CO_2_ insufflation, subxiphoid anterior pericardial access is obtained. CO_2_, carbon dioxide; RAA, right atrial appendage; RF, radiofrequency. These figures were modified from the original figure of Santangeli *et al*. [21].

For these reasons, the previous technique of perforating the RAA using the back end of a coronary guidewire [17] was modified to an approach employing radiofrequency (RF) energy to facilitate more controlled and reliable RAA perforation [21]. A custom telescopic catheter assembly was used, which consisted of a stiff 0.014-inch guidewire (Asahi Astato, Asahi Medical, which has an exposed metal tip and insulated body) inside a 1.8-Fr coronary microcatheter (Terumo FineCross, Terumo Medical) delivered within a 4-Fr hydrophilic angled catheter (Glidecath, Terumo Medical). By navigation with the 4-Fr hydrophilic angled catheter, the perforation site was aimed at the anterior aspect of the distal third of the RAA body to maximize the distance from the perforation site, the parietal pericardium, the epicardial RV, and the right coronary artery. The proximal insulation of the guidewire was shaved off using a surgical blade to expose the metal part of the guidewire. This allows us to deliver RF energy to the metal tip of the guidewire. The tip of the guidewire was exposed from the distal end of the microcatheter by 1–2 mm. The proximal exposed metal end of the guidewire was connected to a hemostat, and a short burst of RF energy was delivered (<1 s, 20–30 W in “cut mode”) with an electrocautery on the hemostat while maintaining the contact of the microcatheter/guidewire assembly to the targeted RAA site. Pictures of the custom-made catheter assembly and the metal exposure at the back end of the guidewire were shown in Fig. 7 (Ref. [21]). When perforating RAA, efforts should be made to avoid excessive guidewire advancement during RF delivery to minimize the risk of collateral damage. After delivering the short RF burst, the guide wire was advanced into the pericardial space (Fig. 8, Ref. [21]). The remainder of the procedure was performed as previously described. Recently, this technique was further modified to utilize a Y-connector with a hemostatic valve attached to the back end of the microcatheter. This allows us to lock the guidewire and microcatheter together and maintain the forward force for effective RF energy delivery while perforating a thick RAA wall.

## 7. Potential Complications Specific to CO_2_ Insufflation via RAA

**Fig. 9.**
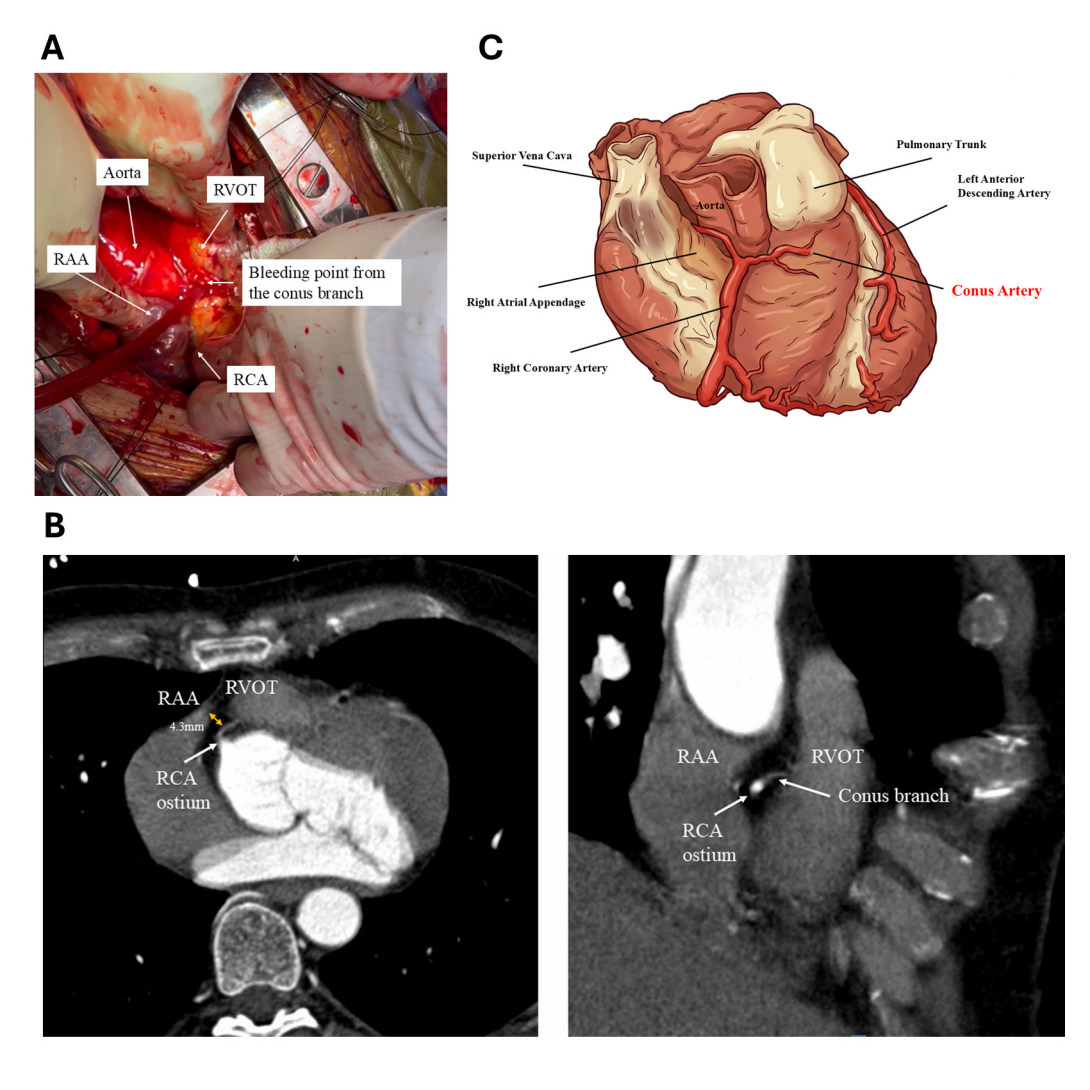
**Conus branch perforation during intentional RAA perforation for CO_2_ insufflation**. (A) Arterial bleeding was identified from the conus branch overlying the fat layer of the RVOT. The bleeding site was sutured, resulting in hemostasis. This figure was modified from the original figure of Higuchi *et al*. [22]. (B) Coronary computed tomography (CT) scan demonstrating the anatomical relationship between the RAA, RVOT, and the conus branch. Axial view (left). RAO view (right). These images highlight the close proximity of the conus branch to the RAA, which is seen overlying the RVOT. This figure was modified from the original figure of Higuchi *et al*. [22]. (C) An image of the course the conus branch and its adjacent structures. This figure was created by FigureLabs. SVC, superior vena cava; RCA, right coronary artery; PT, pulmonary trunk; LAD, left anterior descending; RAA, right atrial appendage; RVOT, right ventricular outflow; RAO, right anterior oblique.

Although the RF-assisted RAA perforation approach for CO_2_ insufflation has been shown to be effective and safe, some risks should be noted. An unforeseen but serious complication associated with the RF-assisted RAA perforation approach was recently reported [22]. In this case, surgical exploration was required due to uncontrolled hemorrhagic pericardial effusion following RF-assisted RAA puncture. This revealed bleeding from a small conus artery (Fig. 9A, Ref. [22]), which was successfully managed by suturing the bleeding point. The proximity of the RAA to the conus branch was later confirmed by CT imaging (4.3 mm away, Fig. 9B, Ref. [22]). The schematic course of the conus artery branch arising from the right coronary artery is illustrated (Fig. 9C). Due to anatomical variability in the right coronary artery and its branches, preprocedural coronary CT imaging may help identify such risks.

## 8. Summary of Each Technique of Epicardial Access

The technique of epicardial access was discussed from the initial era to the contemporary methods. The pros and cons of each method are summarized in Table 1.

**Table 1.** **Pros and cons of each epicardial access technique**.

	Pros	Cons
Conventional technique	∙ Widely adopted	∙ High complication rate
	∙ Fewer steps	
	∙ Shorter procedure time when successful	
Needle-in-needle technique	∙ Less traumatic and forgiving even when the RV is punctured	∙ More steps
		∙ Less feeling of “popping sensation” when entering the pericardial space
SAFER approach	∙ Refinement of the conventional approach	∙ Usage of more contrast in the RV angiogram
	∙ Better visualization of the heart border by RV angiogram	∙ Need for a dedicated injector for RV angiogram
	∙ Safer approach by respiration control	∙ More steps and time
		∙ The additional safety benefit over the conventional approach remains unclear
CO_2_ insufflation through the coronary sinus exit	∙ Safer than other conventional approaches by separating the pericardium	∙ Significantly more steps and time compared with other techniques
		∙ Need for CO_2_ canister
		∙ Potential dissection of the coronary sinus
		∙ Potential dislodgement of the coronary sinus lead
CO_2_ insufflation with RF-assisted RAA exit	∙ Safer than other conventional approaches by separating the pericardium	∙ Significantly more steps and time compared with other techniques
	∙ More straightforward than the coronary sinus exit	∙ Need for CO_2_ canister
		∙ Potential damage to the right coronary artery branch
		∙ Potential interference with the pacemaker lead in RAA

## 9. Future Directions in Epicardial Access: Expanding the Role of CO_2_ Insufflation Techniques

One of the significant advantages of the CO_2_ insufflation technique is the transition from the “blind” puncture to a “guided entry” into a distended, visible anatomical compartment. This fundamental change not only improves safety but also enables the diagnosis of pericardial adhesions. The safety of this technique is provided by the Epi-CO_2_ registry [19] and the comparison with the conventional method [8]. Also, the recent publication of RF-assisted RAA exit for CO_2_ insufflation showed its safety in a single-center experience [21]. Although the proven safety of this method, CO_2_ insufflation has still not been widely adopted due to the significant procedure complexity.

Moving forward, several future directions are likely to broaden the adoption of CO_2_-based access.

∙ Training and simulation: As the barrier to the adoption is largely from the procedure complexity, structured training is needed, such as simulation platforms for CO_2_-guided techniques in risk-free settings.

∙ Expanding indications: Although the current use is largely limited to VT ablation, CO_2_-assisted epicardial access has potential applications in several other procedures. These include epicardial device implantation, such as minimally invasive left atrial appendage closure and epicardial pacemaker lead implantation.

∙ Innovation of dedicated device: A recent preclinical evaluation of a dedicated 1.8F RF-tip microcatheter with a balloon tip demonstrated the safety of RAA perforation and delivery of CO_2_ to the pericardial space [23]. Future development of such dedicated devices may further enable us to reduce the number of procedural steps and operator dependency.

## 10. Conclusion 

Epicardial ablation has become a vital tool for the ablation of VT, particularly in patients with NICM and arrhythmogenic cardiomyopathy. Traditional subxiphoid access techniques, although effective, are associated with significant procedural risks, especially in patients with prior cardiac interventions or abnormal anatomy. Therefore, new techniques, such as the needle-in-needle technique and the SAFER approach, have been adopted to mitigate the risk. CO_2_ insufflation represents a paradigm shift in epicardial access. Several technical refinements and device innovations are still needed to widely adopt this technique.

## Abbreviations

VT, ventricular tachycardia; ICM, ischemic cardiomyopathy; NICM, non-ischemic cardiomyopathy; ARVC, arrhythmogenic right ventricular cardiomyopathy; MRI, magnetic resonance imaging; AP, anteroposterior; LAO, left anterior oblique; RAO, right anterior oblique; LIMA, left internal mammary artery; CO_2_, carbon dioxide; RAA, right atrial appendage; RF, radiofrequency; CRT-D, cardiac resynchronization therapy with a defibrillator.
